# {2-[(1,3-Benzo­thia­zol-2-yl)meth­oxy]-5-bromo­phen­yl}(phen­yl)methanone

**DOI:** 10.1107/S1600536813014086

**Published:** 2013-05-31

**Authors:** K. N. Venugopala, Susanta K. Nayak, B. Odhav

**Affiliations:** a Department of Biotechnology and Food Technology, Durban University of Technology, Durban 4001, South Africa; b Equipe Chimie du Solide et Matériaux, UMR 6226 Institut des Sciences, Université de Rennes 1, Campus de Beaulieu, Avenue du Général Leclerc, 35042 Rennes Cedex, France

## Abstract

In the title compound, C_21_H_14_BrNO_2_S, the dihedral angle between the planes of the benzo­thia­zole and phenyl­methanone groups is 63.4 (2)°. In the crystal, pairs of C—H⋯N hydrogen bonds link the mol­ecules to form inversion dimers, which are further linked by C—H⋯O inter­actions into chains along the *c* axis. C—H⋯π and π–π inter­actions [centroid–centroid distance = 3.863 (1) Å] further stabilize the mol­ecular assembly.

## Related literature

For background to the applications of benzo­thia­zole derivatives, see: Kelarev *et al.* (2003 ▶); Rana *et al.* (2007 ▶); Telvekar *et al.* (2012 ▶); Saeed *et al.* (2010 ▶).
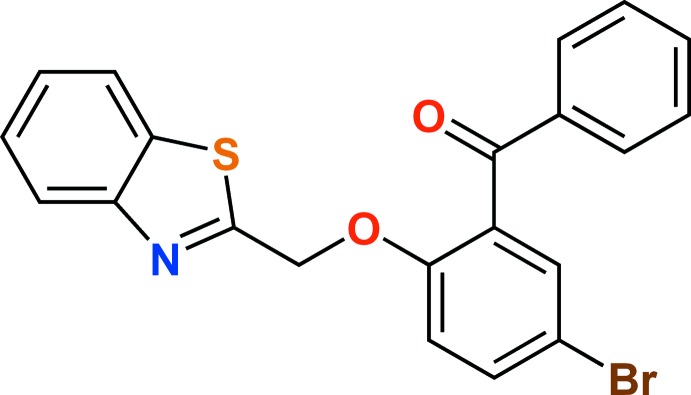


## Experimental

### 

#### Crystal data

C_21_H_14_BrNO_2_S
*M* *_r_* = 424.30
Monoclinic, 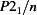

*a* = 15.1475 (6) Å
*b* = 7.6501 (3) Å
*c* = 15.8339 (6) Å
β = 102.105 (3)°
*V* = 1794.03 (12) Å^3^
*Z* = 4
Mo *K*α radiation
μ = 2.42 mm^−1^
*T* = 292 K
0.23 × 0.21 × 0.18 mm

#### Data collection

Oxford Diffraction Xcalibur (Eos, Nova) diffractometer
Absorption correction: multi-scan (*CrysAlis PRO*; Oxford Diffraction, 2009 ▶) *T*_min_ = 0.606, *T*_max_ = 0.670
19116 measured reflections
3525 independent reflections
1971 reflections with *I* > 2σ(*I*)
*R*_int_ = 0.088

#### Refinement

*R*[*F* ^2^ > 2σ(*F* ^2^)] = 0.054
*wR*(*F* ^2^) = 0.120
*S* = 0.98
3525 reflections
235 parameters
H-atom parameters constrained
Δρ_max_ = 0.34 e Å^−3^
Δρ_min_ = −0.32 e Å^−3^

### 

Data collection: *CrysAlis PRO* (Oxford Diffraction, 2009 ▶); cell refinement: *CrysAlis PRO*; data reduction: *CrysAlis PRO*; program(s) used to solve structure: *SHELXS97* (Sheldrick, 2008 ▶); program(s) used to refine structure: *SHELXL97* (Sheldrick, 2008 ▶); molecular graphics: *ORTEP-3 for Windows* (Farrugia, 2012 ▶) and *Mercury* (Macrae *et al.*, 2008 ▶); software used to prepare material for publication: *PLATON* (Spek, 2009 ▶) and *PARST* (Nardelli, 1995 ▶).

## Supplementary Material

Crystal structure: contains datablock(s) global, I. DOI: 10.1107/S1600536813014086/ff2107sup1.cif

Structure factors: contains datablock(s) I. DOI: 10.1107/S1600536813014086/ff2107Isup2.hkl

Supplementary material file. DOI: 10.1107/S1600536813014086/ff2107Isup3.cml

Additional supplementary materials:  crystallographic information; 3D view; checkCIF report

## Figures and Tables

**Table 1:** Hydrogen-bond geometry (Å, °) *Cg*1 is the centroid of the S1/C1/C6/N1/C7 ring.

*D*—H⋯*A*	*D*—H	H⋯*A*	*D*⋯*A*	*D*—H⋯*A*
C21—H21⋯N1^i^	0.93	2.55	3.398 (6)	152
C5—H5⋯O2^ii^	0.93	2.61	3.505 (6)	161
C20—H20⋯*Cg*1^iii^	0.93	2.68	3.459 (4)	142

Symmetry codes: (i) 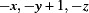; (ii) 
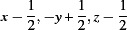
; (iii) 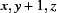.

**Fig. 1.**
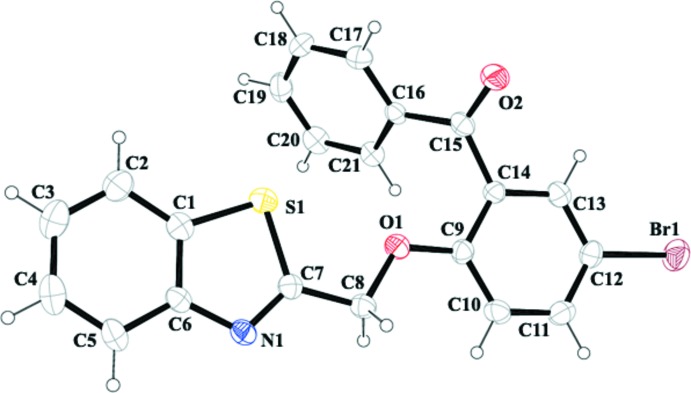
Molecular structure shows the atom labelling scheme with displacement ellipsoids for non-H atoms at 30% probability level, hydrogen atoms are arbitrary circles.

**Fig. 2.**
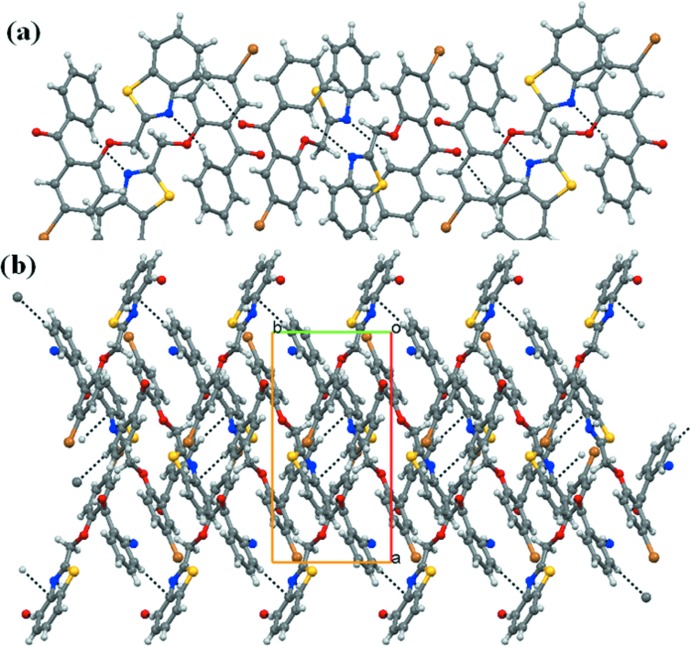
(a) The dimer formation (C—H···N bonds) and their interaction by C—H···O hydrogen bonds. (b) additional C—H···π and π–π interactions stabilize the criss-cross molecular assembly; view along the c axis.

## supplementary crystallographic information

### Comment

Substituted benzothiazole derivatives have been reported to exhibit various pharmacological properties such as analgesic, antibacterial, antifungal, antidepressant, antitumor, antihypertensive, anthelmintic, and herbicidal activity (Kelarev *et al.*, 2003). However, the variety of biological features of new benzothiazole derivatives is of great scientific interest (Telvekar *et al.*, 2012; Saeed *et al.*, 2010). Here, we report the single-crystal structure of the title compound.

The title compound prefers the conformation with the dihedral angle 63.4 (2)° between the planes of benzothiazole and phenylmethanone group (Fig. 1). The weak C—H···N hydrogen bonds lead to dimer formation, whereas C—H···O hydrogen bonds connect the molecules into infinite chains (Fig. 2a), which leads to formation of layers parallel to (-101). Further, the C—H···π interactions involving the five membered ring S1/C1/C6/N1/C7 and π–π [*Cg*2···*Cg*3 = 3.863 (1) Å, *Cg*2 is the centroid of the six membered ring C9—C14 and *Cg*3 is the centroid of the six membered ring C16—C21] stabilize the criss-cross molecular assembly (Fig 2 b).

### Experimental

To a mixture of (2-chloromethyl)benzo[*d*]thiazole (1 mmol) and (5-bromo-2-hydroxyphenyl)(phenyl)methanone (1 mmol) in dry THF, dry potassium carbonate (1 mmol) was added and the reaction mixture was stirred at room temperature for 14 h. The reaction mixture was concentrated to remove the solvent, diluted with ethyl acetate, washed with water, brine solution and dried over anhydrous sodium sulfate. The organic layer was concentrated to yield a residue which was purified by column chromatography using ethyl acetate and n-hexane as eluent (7:3, Rf = 0.71) to afford the product in 77% as a white solid (m. p. 407 (2) K). Suitable crystals for single-crystal X-ray study were obtained from acetonitrile solvent using slow evaporation technique at room temperature.

### Refinement

All H atoms were positioned geometrically and refined using a riding model with *U*_iso_(H)= 1.2 *U*_eq_(C).

### Crystal data

**Table:**

C_21_H_14_BrNO_2_S	*F*(000) = 856
*M**_r_* = 424.30	*D*_x_ = 1.571 Mg m^−^^3^
Monoclinic, *P*2_1_/*n*	Melting point: 407(2) K
Hall symbol: -P 2yn	Mo *K*α radiation, λ = 0.7107 Å
*a* = 15.1475 (6) Å	Cell parameters from 350 reflections
*b* = 7.6501 (3) Å	θ = 1.0–28.0°
*c* = 15.8339 (6) Å	µ = 2.42 mm^−^^1^
β = 102.105 (3)°	*T* = 292 K
*V* = 1794.03 (12) Å^3^	Block, colourless
*Z* = 4	0.23 × 0.21 × 0.18 mm

### Data collection

**Table:**

Oxford Diffraction Xcalibur (Eos, Nova) diffractometer	3525 independent reflections
Radiation source: Mova (Mo) X-ray Source	1971 reflections with *I* > 2σ(*I*)
Mirror monochromator	*R*_int_ = 0.088
Detector resolution: 16.0839 pixels mm^-1^	θ_max_ = 26.0°, θ_min_ = 2.6°
ω scans	*h* = −18→18
Absorption correction: multi-scan (*CrysAlis PRO*; Oxford Diffraction, 2009)	*k* = −9→9
*T*_min_ = 0.606, *T*_max_ = 0.670	*l* = −19→19
19116 measured reflections	

### Refinement

**Table:**

Refinement on *F*^2^	Primary atom site location: structure-invariant direct methods
Least-squares matrix: full	Secondary atom site location: difference Fourier map
*R*[*F*^2^ > 2σ(*F*^2^)] = 0.054	Hydrogen site location: inferred from neighbouring sites
*wR*(*F*^2^) = 0.120	H-atom parameters constrained
*S* = 0.98	*w* = 1/[σ^2^(*F*_o_^2^) + (0.0408*P*)^2^] where *P* = (*F*_o_^2^ + 2*F*_c_^2^)/3
3525 reflections	(Δ/σ)_max_ < 0.001
235 parameters	Δρ_max_ = 0.34 e Å^−^^3^
0 restraints	Δρ_min_ = −0.32 e Å^−^^3^

### Special details

**Table:**

Experimental. CrysAlisPro (Oxford Diffraction, 2009) Empirical absorption correction using spherical harmonics, implemented in SCALE3 ABSPACK scaling algorithm.
Geometry. All s.u.'s (except the s.u. in the dihedral angle between two l.s. planes) are estimated using the full covariance matrix. The cell s.u.'s are taken into account individually in the estimation of s.u.'s in distances, angles and torsion angles; correlations between s.u.'s in cell parameters are only used when they are defined by crystal symmetry. An approximate (isotropic) treatment of cell s.u.'s is used for estimating s.u.'s involving l.s. planes.
Refinement. Refinement of *F*^2^ against ALL reflections. The weighted *R*-factor *wR* and goodness of fit *S* are based on *F*^2^, conventional *R*-factors *R* are based on *F*, with *F* set to zero for negative *F*^2^. The threshold expression of *F*^2^ > 2σ(*F*^2^) is used only for calculating *R*-factors(gt) *etc*. and is not relevant to the choice of reflections for refinement. *R*-factors based on *F*^2^ are statistically about twice as large as those based on *F*, and *R*- factors based on ALL data will be even larger.

### Fractional atomic coordinates and isotropic or equivalent isotropic displacement parameters (Å^2^)

**Table:**

	*x*	*y*	*z*	*U*_iso_*/*U*_eq_	
Br1	0.47887 (4)	0.70720 (7)	0.07925 (3)	0.0671 (2)	
S1	−0.04002 (8)	0.33280 (15)	0.17148 (7)	0.0513 (3)	
O1	0.1169 (2)	0.4165 (4)	0.11024 (17)	0.0499 (8)	
N1	−0.0810 (2)	0.1671 (4)	0.0260 (2)	0.0410 (9)	
O2	0.2746 (2)	0.5691 (4)	0.31997 (18)	0.0587 (9)	
C6	−0.1521 (3)	0.1423 (5)	0.0671 (3)	0.0389 (10)	
C15	0.2206 (3)	0.5983 (5)	0.2529 (3)	0.0395 (10)	
C8	0.0664 (3)	0.3039 (5)	0.0468 (3)	0.0424 (11)	
H8A	0.1004	0.1981	0.0423	0.051*	
H8B	0.0539	0.3614	−0.0090	0.051*	
C16	0.1297 (3)	0.6683 (5)	0.2544 (2)	0.0351 (10)	
C21	0.0809 (3)	0.7618 (5)	0.1858 (3)	0.0396 (11)	
H21	0.1031	0.7750	0.1357	0.048*	
C9	0.1982 (3)	0.4790 (5)	0.0998 (3)	0.0397 (10)	
C7	−0.0190 (3)	0.2608 (5)	0.0734 (2)	0.0379 (10)	
C14	0.2497 (3)	0.5708 (5)	0.1688 (2)	0.0368 (10)	
C12	0.3650 (3)	0.6114 (5)	0.0873 (3)	0.0443 (11)	
C4	−0.2920 (3)	0.0222 (6)	0.0832 (4)	0.0662 (14)	
H4	−0.3432	−0.0445	0.0621	0.079*	
C5	−0.2287 (3)	0.0427 (6)	0.0339 (3)	0.0535 (12)	
H5	−0.2364	−0.0086	−0.0204	0.064*	
C18	0.0136 (3)	0.7221 (6)	0.3328 (3)	0.0554 (13)	
H18	−0.0090	0.7088	0.3827	0.066*	
C2	−0.2067 (3)	0.1994 (6)	0.1977 (3)	0.0600 (14)	
H2	−0.1998	0.2520	0.2517	0.072*	
C20	−0.0003 (3)	0.8356 (5)	0.1912 (3)	0.0507 (12)	
H20	−0.0325	0.8996	0.1449	0.061*	
C1	−0.1411 (3)	0.2200 (5)	0.1478 (3)	0.0438 (11)	
C19	−0.0345 (3)	0.8158 (6)	0.2644 (3)	0.0560 (13)	
H19	−0.0898	0.8653	0.2676	0.067*	
C11	0.3151 (3)	0.5212 (6)	0.0191 (3)	0.0494 (12)	
H11	0.3365	0.5058	−0.0313	0.059*	
C17	0.0947 (3)	0.6485 (6)	0.3280 (3)	0.0472 (12)	
H17	0.1266	0.5846	0.3745	0.057*	
C10	0.2324 (3)	0.4533 (5)	0.0261 (3)	0.0470 (12)	
H10	0.1990	0.3892	−0.0195	0.056*	
C13	0.3333 (3)	0.6361 (5)	0.1623 (3)	0.0436 (11)	
H13	0.3682	0.6967	0.2084	0.052*	
C3	−0.2813 (4)	0.0995 (7)	0.1649 (4)	0.0688 (15)	
H3	−0.3252	0.0830	0.1972	0.083*	

### Atomic displacement parameters (Å^2^)

**Table:**

	*U*^11^	*U*^22^	*U*^33^	*U*^12^	*U*^13^	*U*^23^
Br1	0.0546 (4)	0.0762 (4)	0.0768 (4)	−0.0116 (3)	0.0282 (3)	−0.0030 (3)
S1	0.0522 (8)	0.0533 (8)	0.0482 (7)	−0.0063 (6)	0.0098 (6)	−0.0133 (6)
O1	0.045 (2)	0.055 (2)	0.0503 (19)	−0.0147 (16)	0.0117 (15)	−0.0175 (15)
N1	0.038 (2)	0.043 (2)	0.038 (2)	0.0021 (18)	0.0004 (18)	0.0006 (17)
O2	0.050 (2)	0.079 (2)	0.0432 (19)	0.0160 (18)	0.0014 (16)	−0.0005 (17)
C6	0.036 (3)	0.034 (2)	0.042 (3)	0.005 (2)	−0.001 (2)	0.000 (2)
C15	0.041 (3)	0.034 (2)	0.040 (3)	−0.006 (2)	0.001 (2)	0.001 (2)
C8	0.045 (3)	0.040 (3)	0.042 (3)	−0.003 (2)	0.007 (2)	−0.006 (2)
C16	0.033 (3)	0.034 (2)	0.036 (2)	−0.004 (2)	0.002 (2)	−0.0054 (19)
C21	0.044 (3)	0.030 (2)	0.044 (3)	−0.001 (2)	0.006 (2)	0.004 (2)
C9	0.035 (3)	0.039 (3)	0.045 (3)	0.002 (2)	0.009 (2)	−0.003 (2)
C7	0.037 (3)	0.037 (2)	0.038 (3)	0.007 (2)	0.004 (2)	0.004 (2)
C14	0.036 (3)	0.040 (3)	0.034 (2)	0.003 (2)	0.0060 (19)	−0.001 (2)
C12	0.042 (3)	0.038 (3)	0.055 (3)	0.005 (2)	0.014 (2)	0.002 (2)
C4	0.040 (3)	0.059 (3)	0.097 (4)	−0.002 (3)	0.010 (3)	0.008 (3)
C5	0.038 (3)	0.052 (3)	0.065 (3)	−0.003 (2)	−0.001 (2)	−0.004 (2)
C18	0.047 (3)	0.081 (4)	0.043 (3)	−0.002 (3)	0.019 (2)	−0.006 (3)
C2	0.058 (4)	0.062 (3)	0.064 (3)	0.006 (3)	0.020 (3)	−0.003 (3)
C20	0.053 (3)	0.036 (3)	0.059 (3)	0.005 (2)	0.002 (3)	0.001 (2)
C1	0.044 (3)	0.037 (3)	0.048 (3)	0.004 (2)	0.004 (2)	0.006 (2)
C19	0.039 (3)	0.057 (3)	0.072 (4)	0.000 (3)	0.011 (3)	−0.011 (3)
C11	0.053 (3)	0.053 (3)	0.044 (3)	0.006 (3)	0.016 (2)	−0.003 (2)
C17	0.051 (3)	0.053 (3)	0.037 (3)	0.001 (2)	0.007 (2)	0.003 (2)
C10	0.049 (3)	0.043 (3)	0.046 (3)	−0.003 (2)	0.006 (2)	−0.012 (2)
C13	0.041 (3)	0.041 (3)	0.047 (3)	−0.002 (2)	0.005 (2)	−0.002 (2)
C3	0.059 (4)	0.062 (4)	0.094 (4)	0.005 (3)	0.034 (3)	0.005 (3)

### Geometric parameters (Å, º)

**Table:**

Br1—C12	1.903 (4)	C12—C11	1.368 (6)
S1—C1	1.729 (5)	C12—C13	1.383 (5)
S1—C7	1.739 (4)	C4—C5	1.366 (6)
O1—C9	1.363 (4)	C4—C3	1.399 (6)
O1—C8	1.418 (4)	C4—H4	0.9300
N1—C7	1.289 (5)	C5—H5	0.9300
N1—C6	1.382 (5)	C18—C17	1.367 (6)
O2—C15	1.218 (4)	C18—C19	1.374 (6)
C6—C1	1.388 (6)	C18—H18	0.9300
C6—C5	1.395 (5)	C2—C3	1.373 (6)
C15—C16	1.482 (5)	C2—C1	1.401 (6)
C15—C14	1.502 (5)	C2—H2	0.9300
C8—C7	1.480 (5)	C20—C19	1.372 (6)
C8—H8A	0.9700	C20—H20	0.9300
C8—H8B	0.9700	C19—H19	0.9300
C16—C21	1.379 (5)	C11—C10	1.382 (5)
C16—C17	1.387 (5)	C11—H11	0.9300
C21—C20	1.373 (6)	C17—H17	0.9300
C21—H21	0.9300	C10—H10	0.9300
C9—C10	1.385 (5)	C13—H13	0.9300
C9—C14	1.393 (5)	C3—H3	0.9300
C14—C13	1.386 (5)		
			
C1—S1—C7	88.2 (2)	C3—C4—H4	119.3
C9—O1—C8	119.5 (3)	C4—C5—C6	118.4 (5)
C7—N1—C6	110.3 (3)	C4—C5—H5	120.8
N1—C6—C1	114.8 (4)	C6—C5—H5	120.8
N1—C6—C5	124.5 (4)	C17—C18—C19	120.3 (4)
C1—C6—C5	120.6 (4)	C17—C18—H18	119.8
O2—C15—C16	120.6 (4)	C19—C18—H18	119.8
O2—C15—C14	118.6 (4)	C3—C2—C1	118.2 (5)
C16—C15—C14	120.8 (4)	C3—C2—H2	120.9
O1—C8—C7	107.9 (3)	C1—C2—H2	120.9
O1—C8—H8A	110.1	C19—C20—C21	120.6 (4)
C7—C8—H8A	110.1	C19—C20—H20	119.7
O1—C8—H8B	110.1	C21—C20—H20	119.7
C7—C8—H8B	110.1	C6—C1—C2	120.6 (4)
H8A—C8—H8B	108.4	C6—C1—S1	110.0 (3)
C21—C16—C17	118.8 (4)	C2—C1—S1	129.4 (4)
C21—C16—C15	121.4 (4)	C20—C19—C18	119.5 (5)
C17—C16—C15	119.7 (4)	C20—C19—H19	120.3
C20—C21—C16	120.3 (4)	C18—C19—H19	120.3
C20—C21—H21	119.9	C12—C11—C10	119.1 (4)
C16—C21—H21	119.9	C12—C11—H11	120.5
O1—C9—C10	124.0 (4)	C10—C11—H11	120.5
O1—C9—C14	116.9 (4)	C18—C17—C16	120.5 (4)
C10—C9—C14	119.0 (4)	C18—C17—H17	119.7
N1—C7—C8	122.1 (4)	C16—C17—H17	119.7
N1—C7—S1	116.6 (3)	C11—C10—C9	121.3 (4)
C8—C7—S1	121.2 (3)	C11—C10—H10	119.3
C13—C14—C9	119.6 (4)	C9—C10—H10	119.3
C13—C14—C15	117.3 (4)	C12—C13—C14	120.1 (4)
C9—C14—C15	123.1 (4)	C12—C13—H13	119.9
C11—C12—C13	120.8 (4)	C14—C13—H13	119.9
C11—C12—Br1	120.0 (3)	C2—C3—C4	120.8 (5)
C13—C12—Br1	119.2 (3)	C2—C3—H3	119.6
C5—C4—C3	121.3 (5)	C4—C3—H3	119.6
C5—C4—H4	119.3		
			
C7—N1—C6—C1	−0.4 (5)	C1—C6—C5—C4	−0.6 (6)
C7—N1—C6—C5	−178.1 (4)	C16—C21—C20—C19	−0.7 (6)
C9—O1—C8—C7	−178.5 (3)	N1—C6—C1—C2	−177.6 (4)
O2—C15—C16—C21	155.4 (4)	C5—C6—C1—C2	0.2 (6)
C14—C15—C16—C21	−21.3 (6)	N1—C6—C1—S1	1.8 (4)
O2—C15—C16—C17	−20.9 (6)	C5—C6—C1—S1	179.5 (3)
C14—C15—C16—C17	162.4 (4)	C3—C2—C1—C6	0.5 (7)
C17—C16—C21—C20	0.9 (6)	C3—C2—C1—S1	−178.7 (4)
C15—C16—C21—C20	−175.5 (4)	C7—S1—C1—C6	−1.9 (3)
C8—O1—C9—C10	6.4 (6)	C7—S1—C1—C2	177.4 (4)
C8—O1—C9—C14	−172.2 (4)	C21—C20—C19—C18	0.5 (7)
C6—N1—C7—C8	178.2 (4)	C17—C18—C19—C20	−0.4 (7)
C6—N1—C7—S1	−1.2 (4)	C13—C12—C11—C10	−0.5 (6)
O1—C8—C7—N1	176.5 (4)	Br1—C12—C11—C10	−179.7 (3)
O1—C8—C7—S1	−4.2 (5)	C19—C18—C17—C16	0.5 (7)
C1—S1—C7—N1	1.8 (3)	C21—C16—C17—C18	−0.7 (6)
C1—S1—C7—C8	−177.5 (3)	C15—C16—C17—C18	175.7 (4)
O1—C9—C14—C13	179.4 (4)	C12—C11—C10—C9	1.8 (6)
C10—C9—C14—C13	0.7 (6)	O1—C9—C10—C11	179.5 (4)
O1—C9—C14—C15	1.6 (6)	C14—C9—C10—C11	−1.9 (6)
C10—C9—C14—C15	−177.1 (4)	C11—C12—C13—C14	−0.6 (6)
O2—C15—C14—C13	−45.6 (5)	Br1—C12—C13—C14	178.6 (3)
C16—C15—C14—C13	131.2 (4)	C9—C14—C13—C12	0.5 (6)
O2—C15—C14—C9	132.2 (4)	C15—C14—C13—C12	178.4 (4)
C16—C15—C14—C9	−50.9 (6)	C1—C2—C3—C4	−0.7 (7)
C3—C4—C5—C6	0.4 (7)	C5—C4—C3—C2	0.3 (8)
N1—C6—C5—C4	176.9 (4)		

### Hydrogen-bond geometry (Å, º)

Cg1 is the centroid of the S1/C1/C6/N1/C7 ring.

**Table:**

*D*—H···*A*	*D*—H	H···*A*	*D*···*A*	*D*—H···*A*
C21—H21···N1^i^	0.93	2.55	3.398 (6)	152
C5—H5···O2^ii^	0.93	2.61	3.505 (6)	161
C20—H20···*Cg*1^iii^	0.93	2.68	3.459 (4)	142

Symmetry codes: (i) −*x*, −*y*+1, −*z*; (ii) *x*−1/2, −*y*+1/2, *z*−1/2; (iii) *x*, *y*+1, *z*.
